# The Ubiquitin Proteasome System as a Double Agent in Plant-Virus Interactions

**DOI:** 10.3390/plants10050928

**Published:** 2021-05-06

**Authors:** Ullrich Dubiella, Irene Serrano

**Affiliations:** 1 KWS SAAT SE & Co. KGaA, Grimsehlstraße 31, 37574 Einbeck, Germany; ullrich.dubiella@kws.com; 2 Albrecht-von-Haller-Institute for Plant Sciences, Georg-August-University Göttingen, 37077 Göttingen, Germany

**Keywords:** plant, virus, defence responses, UPS, arms race

## Abstract

The ubiquitin proteasome is a rapid, adaptive mechanism for selective protein degradation, crucial for proper plant growth and development. The ubiquitin proteasome system (UPS) has also been shown to be an integral part of plant responses to stresses, including plant defence against pathogens. Recently, significant progress has been made in the understanding of the involvement of the UPS in the signalling and regulation of the interaction between plants and viruses. This review aims to discuss the current knowledge about the response of plant viral infection by the UPS and how the viruses counteract this system, or even use it for their own benefit.

## 1. Introduction

The ability of plants to survive stresses relies heavily on proteome plasticity via post-translational modifications (PTMs) to regulate protein function. These modifications determine diverse fates of target proteins and enable cells to rapidly and specifically respond to stimuli, avoiding time- and energy-consuming de novo protein synthesis. Reversible protein modification with ubiquitin, ubiquitination, is a key regulatory mechanism that controls diverse aspects of plant biology. Ubiquitination involves the addition of covalently attached ubiquitin residues to substrates in a stepwise cascade: First, an E1 (ubiquitin-activating enzyme) “activates” the ubiquitin by forming a high-energy thioester bond to a ubiquitin moiety, which is then transferred onto the active site of a cysteine residue of an E2 (ubiquitin-conjugating enzyme). Finally, the E2 partners with an E3 (ubiquitin-ligase) and transfers ubiquitin to a lysine residue of the target substrate [1,2]. Compared with other eukaryotes, plant genomes encode a large number of ubiquitin proteasome (UPS) components. In fact, the Arabidopsis genome encodes more than 1600 proteins involved in the ubiquitin-related pathway, underlining the importance of the ubiquitin-related pathway in diverse cellular processes. Most of these genes (>1400) encode putative E3-ubiquitin ligases that are responsible for substrate specificity, since they define the substrates for ubiquitination [1,2,3]. E3 ubiquitin-ligase enzymes are the best characterized components of the ubiquitination cascade, and they are classified into four different protein families (HECT (homologous to E6-associated protein C-terminus), RING (really interesting new gene), U-Box, and CRL (Cullin-RING ligases)), depending on their structural and functional features [1].

Although the most recognized function of ubiquitination involves linkage via Lys48 and subsequently protein degradation through the ubiquitin/26S proteasome system, polyubiquitination via other lysines or Met1 confers non-proteolytic function over the ubiquitinated protein, such as endocytosis, autophagy, protein–protein interaction, protein trafficking or DNA repair [1,4,5,6,7]. This is possible because each linkage type has a distinct three-dimensional topology that results in specific interactions and/or regulation of different biological functions [8,9,10]. In addition, it is now known that there is also reversible ubiquitination, mediated by deubiquitinases (DUBs), which can direct non proteolytic events that affect the regulation of transcription, chromatin structure, and vesicular trafficking [11].

To effectively infect plants, pathogens need to overcome a multi-layered plant innate immune system, coordinated by several subcellular compartments and interactions between these compartments, which efficiently detect potential pathogens and initiate a defence response. The first line of this defence is initiated during the early phases of pathogen infection by the detection of conserved microbial- or pathogen-associated molecular patterns (MAMPs or PAMPs). This recognition is achieved by plant extracellular pattern-recognition receptors (PRRs), leading to PAMP-triggered immunity (PTI) [12]. PTI is a basal immune status that is effective against a broad spectrum of pathogens. Pathogens have evolved ways to circumvent this defence by producing virulence effectors that suppress crucial host PTI regulators, thereby counteracting plant defences, causing effector-triggered susceptibility (ETS). As a consequence, plants have gained the ability to recognise these effectors through resistance (R) proteins, leading to a fast, specific, and effective form of resistance called effector-triggered immunity (ETI). ETI is frequently associated with the development of the hypersensitive response (HR), a localised programmed cell death that prevents the spread of the pathogen beyond the site of infection [12]. The UPS has been shown to play a crucial role during the regulation of plant immune signalling, being involved in all steps of plant defence responses from pathogen perception to regulation of downstream signalling [4,6,13,14,15].

Viruses are small, intracellular, obligate, biotrophic parasites that rely on their host in nearly every aspect of their life cycle as they do not possess their own replication machinery. Although historically viruses have been considered non-PAMP-coding pathogens [16], several lines of evidence suggest that PTI may play a role in both compatible and incompatible plant-virus interactions [17,18]. Besides the role that plant innate immunity may play in defence against viruses, other active defences include RNA silencing, translational repression, protein degradation, and direct interaction and inhibition of viral proteins [16,19,20].

Because of their intracellular nature, viruses interact very closely with host cellular compartments, which makes direct antiviral treatment difficult. Reports show that exogenous application of dsRNA can indeed confer resistance against RNA viruses, but the application, especially under open field conditions, still faces several problems, such as the delivery into plant cells and stability of the dsRNA [21,22]. Therefore, so far, the most effective ways to control viruses in the field are genetic resistance by breeding for resistance genes from wild relatives or chemical control of the vector. The most common vectors for plant viruses are insects of the Hemipteran order, which transmit the virus from plant to plant within the local population, but also to geographic distant locations [23]. Some examples for genetic virus resistance are Rz1 and Rz2 in sugar beet (*Beta vulgaris*), which confer resistance against beet necrotic yellow vein virus (BNYVV) [24,25]; Ty-1, TY-2, and Ty-3 from tomato for resistance against tomato yellow leaf curl virus (TYLCV) [26]; and CMV1 from melon against cucumber mosaic virus (CMV) [27]. However, creating genetic resistance against viruses by breeding is not always possible. Finding and confirming potential resistance genes from natural populations is a very labour intensive and costly endeavour. Therefore, the use of fungicides or insecticides, such as neonicotinoids, is still crucial for virus control. However, neonicotinoids were found to harm insects in general, bees in particular, and are banned for use in the field in the European Union (EU) (Regulation (EU) No. 485/2013). Furthermore, it is to be expected that agrochemicals in general will be under stronger regulation in the near future. This will make chemical vector control less efficient and, thus, it is to be expected that viral infections will become a big problem, especially for field crops.

Viruses succeed during plant infection thanks to their ability to reprogram the host cell. Although viruses usually possess very small genomes, and thus a very limited set of encoded proteins, one protein often does have more than just one function [28]. Posttranslational modifications, such as phosphorylation, acetylation, glycosylation, and ubiquitination are crucial to fine tuning the different functions of the viral proteins and re-localising them from one cellular compartment to another [29,30,31,32].

While there is an increasing body of evidence about the importance of the UPS during the plant–virus interaction, it is becoming clear that the plant UPS acts as a two-edged sword during viral pathogenesis, alternatively impairing and facilitating viral infection [33,34]. While, on the one hand there are several studies describing the antiviral function of the UPS in plants, on the other hand, more and more studies show that the host UPS has a positive effect on viral replication. These interactions serve to regulate virus infection and/or promote virus replication and movement, but they also modulate the levels of RNA accumulation to ensure successful biotrophic interactions [34]. Here, we summarize the current knowledge about the arms-race between plants and viruses to use the UPS for their own respective benefit during infection.

## 2. The Role of the UPS in Plant Defences against Viruses

### 2.1. UPS-Dependent Activation of Viral-Induced Defence Responses

After an early report showing that tobacco plants expressing a ubiquitin variant with a Lys to an Arg change had an altered response to viral infection [35], an increased number of genes related to the UPS have been shown to be involved in regulating viral infection [4,16,17,33].

Two *Nicotiana tabacum* ubiquitin-activating enzymes (E1), NtUBA1 and NtUBA2, were found to be upregulated during tobacco mosaic virus (TMV) and tomato mosaic virus (ToMV) infections [36], suggesting the involvement of the UPS in plant defence against these viruses. A later report showed that silencing of NtUBA1 promotes tomato yellow leaf curl virus (TYLCSV) infection in *N. tabacum* [37]. This indicates that this UPS component is crucial in either suppressing viral infection or that it is a target of a viral pathogenicity factor. Similarly, silencing of tomato 26S proteasome subunit SlRPT4a caused the conversion of tolerant plants into susceptible plants after tomato leaf curl Delhi virus (ToLCNDV) infection [35]. Furthermore, functional characterization linked SlRPT4 interference of ToLCNDV infection to the activation of HR [38].

One of the three major signalling modules that function in early HR signalling comprises an adaptor protein, SUPPRESSOR OF THE G2 ALLELE OF SKP1 (SGT1), which physically interacts with REQUIRED FOR MLA12 RESISTANCE1 (RAR1), HEAT SHOCK PROTEIN90 (HSP90), and R proteins [39,40,41]. SGT1 interacts with multiple E3-ubiquitin-ligase components, such as S PHASE KINASE-ASSOCIATED PROTEIN1 (SKP1) and CULLIN1 in the CRL complex and components of the COP9 signalosome (CSN) [42]. During TMV infection of *Nicotiana benthamiana*, both SGT1 and RAR1 interact with CSN3 and CSN8 to mediate the N gene resistance against TMV; plants knocked out for expression of NbSGT1, NbSKP1, or the CSN are compromised in resistance against TMV [43,44].

Beside its proteolytic activities, the 20S proteasome also contains ribonuclease (RNase) activity and this activity appear to impact virus accumulation. The UPS RNase activity, previously detected in 20S proteasome preparations from *Helianthus annuus*, has been shown to specifically target Lettuce mosaic virus (LMV) and TMV RNAs in vitro [45].

These examples support the idea that the UPS is a crucial player in the molecular dialogue between plant and virus, and, moreover, represents an integral part of the plant antivirus defence system.

### 2.2. Targeting of Viral Proteins by the UPS

The first viral proteins found to be ubiquitinated were TMV coat proteins (CPs) [46]. This work suggested for the first time that ubiquitination could be part of the plant responses to viral infection. Since then, a growing body of evidence has shown that, in fact, the UPS is able to directly target different viral proteins, affecting viral infection and disease symptoms (Table 1).

Many plant viruses encode for specialized movement proteins (MPs) to facilitate virus trafficking from one infected cell to neighbouring cells. Degradation of MPs by the UPS may be considered an effective host defence pathway against viral infection, since MPs are crucial for the spreading of viruses and may play a role in suppression of RNA silencing [47,48]. The first MP reported to be degraded in vivo was TMV 30K MP. The use of plant proteasome inhibitors led to an increased accumulation of TMV 30K MP, suggesting that targeting and subsequent degradation of this protein by the UPS may play a role in regulation of the systemic spread of the virus and in the damage caused by accumulation of MP in the endoplasmic reticulum [49]. Additionally, Gillespie et al. [50] showed that impairment of TMV MP degradation by disruption of the UPS improved viral transport. Turnip yellow mosaic virus (TYMV) MP 69K is also specifically recognized as a substrate for polyubiquitination and is subsequently degraded by the proteasome [51]. Potato virus X (PVX) triple gene block protein 3 (TGBp3) is required for virus cell-to-cell movement and is targeted for degradation by the host proteasome [52]. Another example is potato leafroll virus (PLRV) MP 17K, which has been shown to accumulate in aggresomal-like structures upon inhibition of the host proteasome [53].

A way of limiting virus infection is to limit virus replication by targeting proteins crucial for this process. Viruses depend on many critical interactions with the host in order to assemble viral replication complexes, thus, modification of complex subunits by selective ubiquitination is likely to interfere with proper viral replication [33]. A *N. benthamiana* RING E3 ligase (NbUbE3R1) has been shown to be involved in the accumulation of bamboo mosaic virus (BaMV), since silencing of this gene induces an increase in virus accumulation. Further analysis identified the replicase of BaMV as the possible substrate of NbUbE3R1, which resulted in down-regulating BaMV replication [54].

Another recent example of an active defence role of the UPS against viruses is the degradation of the silencing suppressor p3 from rice stripe virus (RSV) [55]. Although Ub was the first polypeptide modifier described [1,3,56,57], the discovery of Ub-like modifiers (UBLs) led to a constellation of peptide tags that can be reversibly attached to substrates [58,59,60]. It has been shown that a ubiquitin-like protein (UBL5) interacts with p3 directly and that silencing of UBL5 leads to an accumulation of p3 in *N. benthamiana*. On the other hand, overexpression of UBL5 inhibited RSV infection [55].

The yeast Rsp5p is a member of the Nedd4 family of E3 ubiquitin ligases and it blocks tomato bushy stunt virus (TBSV) replication by interacting with the RNA-binding sites on two replication proteins (p33 and p92pol) [61,62,63]. Since the ability to block viral replication was dependent on the Rps5p WW protein interaction domain, but was independent of the HECT domain, interaction rather than ubiquitination plays a role in virus replication [61]. The fact that purified recombinant Rsp5p also inhibited RNA replication in a cell-free TBSV replication assay, underlines the complexity of Rsp5p inhibitory function [61]. Similar proteins with a WW domain could have similar viral inhibitory effects in planta, opening the possibility of using the interaction mediated by WW domains as a potential antiviral strategy [61,64].

The initiation of replication of positive-strand RNA viruses relies on viral RNA-dependent RNA polymerase (RdRP). Camborde et al. [65] showed that RdRP (termed 66k) is a target of the UPS at rather late infection time points, supporting the idea that proteasomal degradation may constitute an essential way of regulating viral replication and systemic spreading.

The βC1 protein of tomato yellow leaf curl China virus (TYLCCV) is a determinant for symptom development during viral infection. Interaction of βC1 with tobacco RIN E3 ubiquitin-ligase NtRFP1 mediates its ubiquitination and degradation, attenuating virus infection and symptom development [66]. βC1 induces severe stunting and leaf curling when overexpressed in transgenic lines [67]. Plants overexpressing NtRFP1 have attenuated disease symptoms, while NtRFP1 knock-down plants develop more severe symptoms. However, in both cases viral DNA accumulation is not affected [66], indicating that in this case the UPS is able to mitigate disease symptoms rather than to prevent disease.

A recent report has provided evidence for the role of plant ubiquitin extension proteins (UEPs), which are the main source of ubiquitin, in disease resistance against TMV and CMV. Plants silenced for UEP1 exhibited intensive cell death and increased disease resistance, probably due to the increased accumulation of UPS targets due to the limitation of ubiquitin [68].

Taken together these examples (summarize in Table 1) show how effective the UPS can be at limiting virus replication, spread, or virus dependent symptom development, by targeting viral proteins for degradation or inhibiting their function.

**Table 1:** Viral proteins targeted by the UPS.

Virus	Virus Type	Target	Protein Type	Proposed Outcome	Reference
TMV	+ssRNA	CPs	coat proteins	host defence response?	[46]
TMV	+ssRNA	30K	movement protein	prevention of systemic virus spread	[49,50]
TYMV	+ssRNA	69K	movement protein	ensuring protein transient accumulation?	[51]
PVX	+ssRNA	TGBp3	movement protein		[52]
PLRV	+ssRNA	17K	movement protein		[53]
BaMV	+ssRNA	replicase	replicase	downregulation of viral replication	[48]
RSV	-ssRNA	p3	silencing suppressor	inhibition of viral infection	[55]
TBSV	+ssRNA	p33	replicase	blockage of viral replication	[62,63,69]
TBSV	+ssRNA	p92	replicase	inhibition of viral replication	[62,63,69]
TYMV	+ssRNA	66k	polymerase		[70]
TYLCCV	ssDNA	βC1	pathogenicity determinant	mitigation of disease symptoms	[66,67]

## 3. Using the Host for Your Own Benefit

### 3.1. Active Manipulation of the UPS by Viruses

Proteasomal degradation pathways play a central role in regulating a variety of protein functions by controlling not only their turnover but also the physiological behaviour of the cell. Furthermore, the UPS does not differentiate between ubiquitinated proteins originated from the plant itself or from a virus. This makes this system an attractive target for viruses to manipulate the plant cellular machinery for their own propagation and pathogenesis.

**Figure 1:**
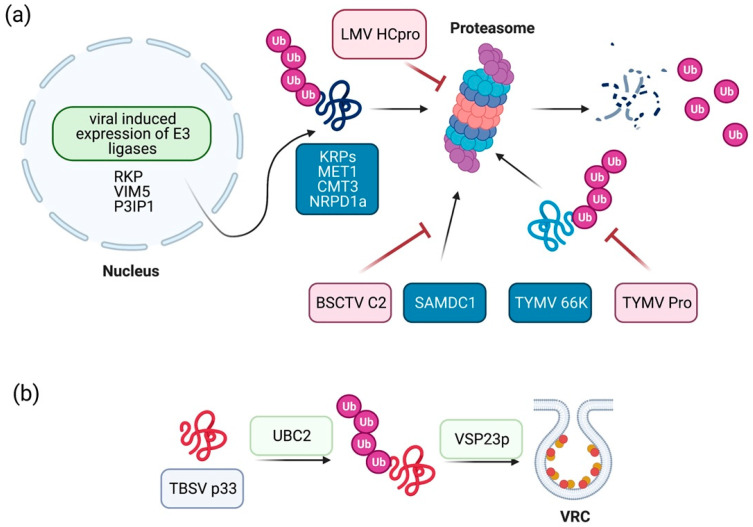
Viral manipulation of the host UPS. (**a**) Viruses can inhibit the function of the proteasome either by direct interaction with its subunits (LMV HCpro) or by induction of the expression of plant E3-Ub ligases (RKP, VIM5, and P3IP1). This induction leads to the subsequent degradation of antiviral host factors. Furthermore, virus proteins (BSCTV C2) can interact directly with host factors (SAMDC1) to prevent its proteasomal degradation, or TYMV Pro can counteract ubiquitination via its deubiquitinase activity. (**b**) Viruses form viral replication complexes (VRC) using membranous structures from the host. TBSV uses the UPS in order to facilitate the formation of VRCs.

Viruses have the possibility to exploit the UPS at different levels (Figure 1). They can (I) induce/inhibit the expression of UPS related genes, or they can induce/inhibit the activity of E3-ubiquitin ligases and associated proteins; (II) directly influence the activity of subunits of the proteasome, and (III) manipulate the ubiquitination status of host proteins. One example of a direct targeting of the host proteasome, considered as part of a general antiviral defence pathway, is the one carried out by HcPro (helper component proteinase) protein from LMV. HcPro is involved in numerous steps of the viral lifecycle, including replication, cell-to-cell movement, and vector transmission [71]. In addition, it is also well described as a silencing protein that suppresses post-transcriptional gene silencing [72,73,74]. This viral protein was shown to interact with different 20S proteasome subunits to inhibit its RNAse activity, and thus lead to viral accumulation [75]. Furthermore, potato virus Y (PVY) HcPro has been shown to interact in vitro and in vivo with three Arabidopsis 20S proteasome subunits (α1, β2, and β5) [76].

Geminiviruses, which possess circular single stranded DNA genomes, replicate in the nucleus of the plant cell, and manipulate several UPS-related mechanisms to promote their own replication. The C4 protein from beet severe curly top virus (BSCTV) induces the transcription of RELATED TO KPC1 (RKP) in *Arabidopsis thaliana*. RKP encodes an E3 ubiquitin ligase that regulates the degradation of the cell-cycle inhibitor proteins KIP-RELATED PROTEINS (KRPs). This, in turn, enhances the cell cycle transition and creates a favourable environment for BSCTV replication [77].

Transcriptional activation of another E3 ubiquitin ligase, VARIAN IN METHYLATION5 (VIM5), was found to be associated with the Rep protein from BSCTV. VIM5 triggers the degradation of the DNA methyltransferases MET1 and DNA (cytosine-5)-methyltransferase (CMT3). Without MET1 and CMT3 present, the circular viral genome is hypomethylated, and the transcription of the C2 and C3 genes is induced [78]. In addition, C2 from BSCTV itself blocks protein degradation by the UPS indirectly to prevent methylation-mediated gene silencing of the circular genomic DNA. Earlier, it was demonstrated that C2 interacts with S-ADENOSYL-METHIONINE DECARBOXYLASE1 (SAMDC1), a susceptibility factor for BSCTV infection, and attenuates its degradation by the proteasome. SAMDC1 catalyses the conversion of S-Adenosyl-methionine (SAM) to decarboxylated S-adenosyl-methionine (dcSAM). SAM is the major methyl donor for methylation processes, including DNA methylation, and dcSAM is considered to be a competitive inhibitor of SAM [79]. Thus, attenuation of the degradation of SAMDC1 will result in higher concentrations of dcSAM with a stronger inhibition of viral DNA methylation.

Furthermore, the C2 protein from another Geminivirus, TYLCV, seems to promote the interaction of whiteflies (the vector for TYLCV) and infected plants [80] by interacting directly with the ubiquitin moiety of ubiquitin-40S ribosomal protein S27a (RPS27a). Although the direct mechanism is not known yet, it is postulated that this interaction is possibly responsible for the block of ubiquitination and subsequent 26S proteasomal degradation of JAZ1, which blocks MYC2 dependent expression of defence-related genes [80].

Another example of viruses using the host UPS to degrade host factors is the induced expression of P3-INDUCIBLE PROTEIN (P3IP1), a U-box type E3 ligase, in rice plants after infection with rice grassy stunt virus (RGSV) or after overexpression of the P3 protein alone. P3IP1 was shown to be a negative regulator for resistance against RGSV by targeting a subunit of the rice Pol-IV (OsNRPD1a) for proteasomal degradation [81]. Interestingly, the same study showed a direct interaction of P3 with the rice NUCLEAR RNA POLYMERASE D1a (OsNRPD1a), although the function of this interaction remains unknown.

RNA plant viruses have a strong need to suppress the activity of their host RNA-silencing machinery in order to promote viral genome replication. The P0 protein from Poleroviruses functions as a silencing suppressor [82,83,84,85,86,87,88] and is responsible for the active degradation of ARGONAUTE1 (AGO1), a central component of the RNA-induced silencing complex (RISC) [89]. Because of initial reports showing that P0 proteins from different viruses interact with the F-BOX PROTEIN S PHASE KINASE-ASSOCIATED PROTEIN1 (SKP1), a member of the SCF E3 ubiquitin complex, and trigger ubiquitination of AGO1 [90,91], it was thought that P0 hijacks the UPS to suppress the plant silencing machinery. In contrast to that conclusion, later findings suggest that P0-triggered AGO1 degradation is insensitive to proteasome inhibitors [92,93]. Moreover, the P0 protein from PLRV does not interact with SKP1, but it is still capable of triggering AGO1 degradation. Zhou et al. and Derrien et al. [94,95] found that autophagy is responsible for AGO1 degradation. Therefore, it seems clear that silencing suppression of Polerovirus P0 proteins does not rely on the UPS. So, the real function of the described P0-SKP1 interaction is still elusive and more research is needed to decipher its real function.

Other viral proteins interacting with components related to the SCF complex are the pathogenicity factor P25 from BNYVV and βC1 from cotton leaf curl multan virus (CLCuMuV). It has been shown that P25 interacts with a KELCH-REPEAT F-BOX FAMILY PROTEIN (FBK) from sugar beet, which in turns interacts with SKP1-LIKE (ASK) [96]. However, to date the real effect of this interaction is still unknown.

Additionally, the βC1 protein from CLCuMuV was found to interact with the tomato ubiquitin conjugating enzyme SlUBC3 [97]; however, silencing of UBC3 in *N. benthamiana* did not show any effect on symptom development or viral DNA accumulation [98]. However, in this last work the authors were able to demonstrate that CLCuMuV βC1 impairs the function of SCF complexes in *N. benthamiana* by direct interaction with SKP1s and that this interaction disrupts the interaction between SKP1 and CUL1.

### 3.2. Using Antiviral Responses for Yourself

It is unclear if the sole role of viral protein degradation by the plant UPS is limiting virus spread and thus promoting host defence. Viruses may well regulate their lifecycle by targeting abnormal or excess proteins for degradation, taking advantage of the host UPS machinery [33]. Although viral proteins can make up to 20% of the total protein mass of a cell, infected cells are able to survive for weeks [99]. In this work the authors could demonstrate that only the misfolded and insoluble CP from TMV is ubiquitinated, but not soluble CP. Later, it was shown that soluble CP from TMV and potato virus A (PVA) can also be ubiquitinated by plant E3 ligases and that they are degraded by the proteasome [100,101]. It is speculated that ubiquitination of CP is the molecular switch that regulates the transition from early-to-late infection stages [101].

In other cases, ubiquitination of viral proteins seems to be a prerequisite for successful viral replication. For TBSV, for example, it could be demonstrated that the replication protein p33 is ubiquitinated by two host factors, Rad6/ubc2 and cdc34p [28,102]. Mutations in either of these two proteins inhibits viral replication, indicating a direct positive role of these proteins in viral replication. Nevertheless, it has been shown that too high levels of p33 ubiquitination are inhibitory for viral replication [28], demonstrating that regulation of the biological function of viral proteins by ubiquitination is a complex and fine-tuned system. Furthermore, Rad6/ubc2 is an important factor for the recruitment of VPS23p to the replication complex [103]. VPS3p is an endosomal sorting complex required for transport (ESCRT) proteins, and ESCRT proteins are necessary to the formation of spherular compartments along membranes, also called viral replication complexes (VRC). Tombusviruses form VRCs, as do all plant RNA viruses, to replicate their genome and assemble new viral particles in an environment protected from exposure to host antiviral mechanisms [69,103,104].

The protein 66k, the RdRP from TYMV, is another example, where ubiquitination and degradation of viral proteins is detrimental to successful viral replication. The 66k accumulates only at low levels within the cell and, as mentioned above, it was demonstrated that 66K is indeed ubiquitinated by host factors [65]. Therefore the host induced ubiquitination and subsequent degradation is very important for a successful TYMV replication. However, to prevent total degradation of 66K and counteract the ubiquitination on at least a small amount of protein, TYMV encodes for a protease protein (TYMVpro) that also functions as a DUB. Furthermore, recently, is has been shown that the deubiquitination activity of TMYVpro is 10–1000-fold lower than that known from other viral deubiquitinating proteins, and it is postulated that this low activity is an evolutionary compromise to keep the 66k levels low in order to ensure proper viral replication [105]. These apparently conflicting results highlight the complexity of the interactions between plant viruses and the UPS pathway.

## 4. Conclusions

Viruses, as intracellular obligate biotrophic pathogens with a very limited amount of self-coded proteins, depend on host factors at every step of their lifecycle. The UPS machinery of plants is an essential part of cellular immunity and can intervene at each step of the viral infection. Viruses, in turn, have developed many ways to not only circumvent UPS-dependent plant defence responses but also to utilize the UPS for their own benefit. They use the UPS to reshape physiological processes or cellular compartments in order to facilitate their own replication or movement. Thus, the UPS is yet another important area of the arms race between viruses and plants, where shutting down or manipulating the UPS by the pathogen is an important factor in determining successful infection or defence.

Since viruses depend heavily on their host, they have an intimate relationship with many host proteins. These host proteins can be divided into two major categories. One includes proteins with antiviral functions, where the interaction aims to suppress plant immunity. The second comprises proteins that are detrimental for successful infection, replication, movement, and/or transmission, where a successful interaction makes the plant susceptible to the virus. Therefore, these genes are called susceptibility genes (S-genes) [106].

In turn, if viruses need these proteins for successful infection, it should be possible to generate virus-resistant plants by either modifying host factors in order to prevent viral protein interactions or by inhibiting the expression of these host factors [106,107]. This leads to the question if factors of the UPS, which are actively manipulated by viruses, can/should be considered as potential susceptibility factors (S-factors) [108].

Genome-editing technologies are rapidly evolving and can be used to disrupt or interfere with virus–host protein–protein interactions in order to create virus resistant plants. Probably the best described example for this kind of resistance is the translation initiation factor eIF4E1 [109]. Design of a synthetic eIF4E1 allele by introducing amino acid changes associated with resistance to Potyvirus in *Pisum sativum*, confers resistance to *A. thaliana* while retaining protein functionality. It is especially important to keep in mind that the UPS has been shown to be a crucial component of plant defence responses, including defence against viruses, and thus the modification of host factors should not be detrimental for the plant. In fact, in some of the examples described knockout mutations are detrimental for virus infection, but in other cases knockout mutations are beneficial for the virus, leading to a higher virus accumulation and a more aggressive disease. This shows that detailed knowledge about the specific virus–host interaction is necessary to engineer variants of the S-factors. In order to exploit the UPS as a potential target to create resistant plants, it is evident that more research is necessary to gain sufficient understanding of the comprehensive and competitive network formed between plants and viruses in this context.
